# Two Immunoglobulin Tandem Proteins with a Linking β-Strand Reveal Unexpected Differences in Cooperativity and Folding Pathways

**DOI:** 10.1016/j.jmb.2011.12.012

**Published:** 2012-02-10

**Authors:** Annette Steward, Qing Chen, Robert I. Chapman, Madeleine B. Borgia, Joseph M. Rogers, Alexsandra Wojtala, Matthias Wilmanns, Jane Clarke

**Affiliations:** 1 Department of Chemistry, University of Cambridge, Lensfield Road, Cambridge CB2 1EW, UK; 2 EMBL Hamburg, Notkestrasse 85, D-22603 Hamburg, Germany

**Keywords:** multidomain, Beta sheet, titin A-band, tandem repeat, protein folding, FNIII, fibronectin type III

## Abstract

The study of the folding of single domains, in the context of their multidomain environment, is important because more than 70% of eukaryotic proteins are composed of multiple domains. The structures of the tandem immunoglobulin (Ig) domain pairs A164–A165 and A168–A169, from the A-band of the giant muscle protein titin, reveal that they form tightly associated domain arrangements, connected by a continuous β-strand. We investigate the thermodynamic and kinetic properties of these tandem domain pairs. While A164–A165 apparently behaves as a single cooperative unit at equilibrium, unfolding without the accumulation of a large population of intermediates, domains in A168–A169 behave independently. Although A169 appears to be stabilized in the tandem protein, we show that this is due to nonspecific stabilization by extension. We elucidate the folding and unfolding pathways of both tandem pairs and show that cooperativity in A164–A165 is a manifestation of the relative refolding and unfolding rate constants of each individual domain. We infer that the differences between the two tandem pairs result from a different pattern of interactions at the domain/domain interface.

## Graphical Abstract

**Figure:**
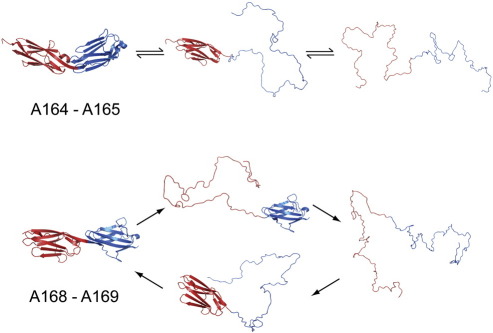

**Highlights:**

► We investigate the stability and folding of titin Ig domains in a multidomain environment. ► Tandem A-band domains in A164–A165 and A168–A169 are linked by a continuous β-strand. ► At equilibrium, A164–A165 exhibits cooperativity, but A168–A169 domains do not interact. ► Modeling using kinetic data shows that an intermediate accumulates in A164–A165. ► Biophysical studies show that these homologous proteins exhibit very different properties.

## Introduction

Many folding studies have concentrated on domains isolated from larger, multidomain proteins rather than on domains linked covalently to their natural neighbors.^1^ In an analysis of all known protein sequences, analyzed in terms of families that have single-domain or multidomain architectures, growth of new single-domain families is low and almost all growth comes from new multidomain proteins.^2^ In the context of a multidomain protein, we therefore need to ask the following questions: Are protein domains stabilized by their neighbors? Are folding and unfolding rates of individual domains altered? How do domains avoid misfolding?

Many proteins, especially in eukaryotes, contain tandem repeats of domains from the same family.^3,4^ The titin I-band is composed of immunoglobulin (Ig) domains and has been experimentally characterized as being extremely flexible.^5–8^ Molecular dynamics simulations also demonstrate how domain/domain arrangements and motions result in tertiary-structure elasticity.^9^ We have previously studied the folding properties of neighboring Ig I-set domains of titin taken from the I-band, I27–I32, as single domains and as tandem dimers and trimers.^10^ These domains act entirely independently, and adjacent domains have very different kinetic properties, suggesting that neighboring domains are very unlikely to unfold during stretching of the muscle. This would be favorable for recovery of titin on relaxation, since misfolding events would be less likely to occur.^11^ In the tandem domain pair FNfn9–FNfn10, consisting of two Ig-like fibronectin type III (FNIII) domains from human fibronectin, each domain was also shown to act independently of its neighbor, with no significant interaction between the pair.^12^ It has been suggested that the independent folding observed in these Ig and FNIII multidomain proteins is a result of the small interface between domains (272 Å^2^ FNfn9–FNfn10).^13^

In some cases, there are significant interactions between the domains in neighboring proteins.^13^ A recent review by Feige *et al.*^14^ describes the folding of Ig domains in IgG antibodies, which can be grouped into three folding categories: those domains that fold autonomously to a monomeric state; those that form an obligate homodimer, controlled by proline isomerization; and the recently discovered template-assisted folding of the CH1 (constant heavy 1) domain, which interacts with the CL (constant light) domain in the intact antibody. One case of particular interest is the 15th, 16th, and 17th spectrin domains, from chicken brain α-spectrin (R15, R16, and R17).^15^ These are three-helix bundle proteins in which the domains are linked by an extended helix that traverses the entire length of both domains. As in Ig and FNIII domains, the interfaces between spectrin domains are relatively small and flexible, but these domains are stabilized by their neighbors and both folding and unfolding rates are affected.^16^ The interactions between these spectrin domains are mediated by the shared helix. In this study, we set out to investigate whether a shared β-strand could have the same effect.

**Fig. 1:**
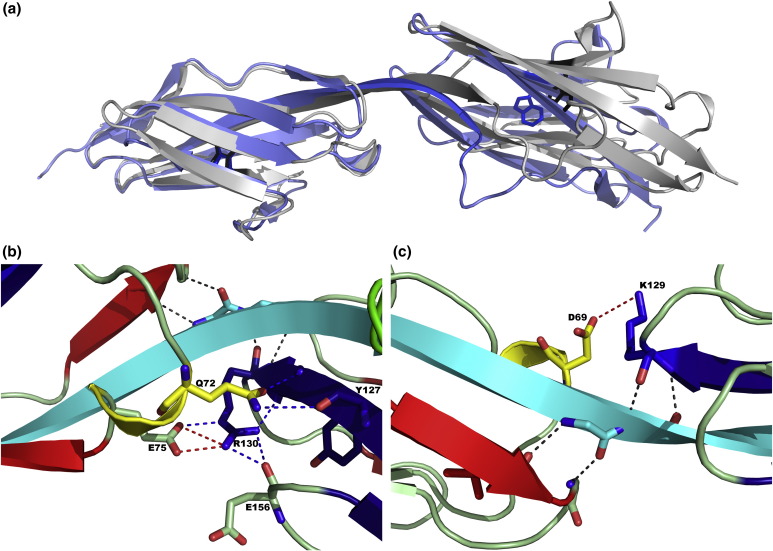
Structures of A164–A165 and A168–A169. (a) Ig tandem A164–A165 (violet; Protein Data Bank code: 3LCY) superimposed to Ig tandem A168–A169 (gray; Protein Data Bank code: 2J8H) showing connecting β-strand between each domain. Each domain has a single, buried Trp residue, shown, which is highly conserved in all Ig domains. These domains have been aligned using the N-terminal domain. Thus, the Trp residue in A164 is superimposed on that of A168. Domains A165 and A169 do not superimpose well, reflecting the different rotation of the domains in the tandem pairs. (b) The domain interface of A164–A165: the N-terminal domain is shown in red, the C-terminal domain is in blue, the connecting β-strand is in cyan, the loops are in light green, and the helical residues are in yellow. Side chains of buried residues Q72, E75, and R130 are involved in a network of interdomain interactions, both hydrogen bonds (blue) and a salt bridge (red). (c) The domain interface of A168–A169: presented in the same way as in (b). There is a surface-exposed salt bridge between D69 and K129 but no other side-chain-mediated hydrogen bonds.

The A-band of titin is made up of Ig and FNIII domains, which are mostly arranged in repeating patterns.^17,18^ A conformational characterization of three FNIII tandems from the A-band shows that these contrast markedly with Ig tandems from the elastic I-band: the FNIII interfaces were found to be conformationally well defined, exhibit limited dynamics, and are largely conserved.^19^ The Ig I-set domains A164, A165, A168, and A169 are located in the A-band segment of titin, N-terminal to the titin kinase domain and M-band.^20,21^ The linker between the two domains is one residue shorter than that between Ig domains in the I-band of titin. Importantly, in the tandem Ig pairs A164–A165 and A168–A169, the C-terminal G-strands of A164 and A168 are connected via a continuous β-strand with the N-terminal A-strands of A165 and A169, respectively (Fig. 1a). Here, we investigate the hypothesis that a continuous β-strand can impart cooperative folding between adjacent Ig domains, as a continuous α-helix does in spectrin R1617.^22^

## Results

### Selecting domain boundaries

In studies of multidomain proteins, it is essential to ensure that the domain boundaries are chosen correctly. Interdomain stabilization might be inferred if domains are cut “too short” when being isolated from a multidomain protein.^12^ We used previous experience from our laboratory and others^10,23,24^ to choose the domain boundaries for the individual Ig domains (see Supplementary Table 1).

### Domains in A164–A165 behave as a single cooperative unit at equilibrium

**Fig. 2:**
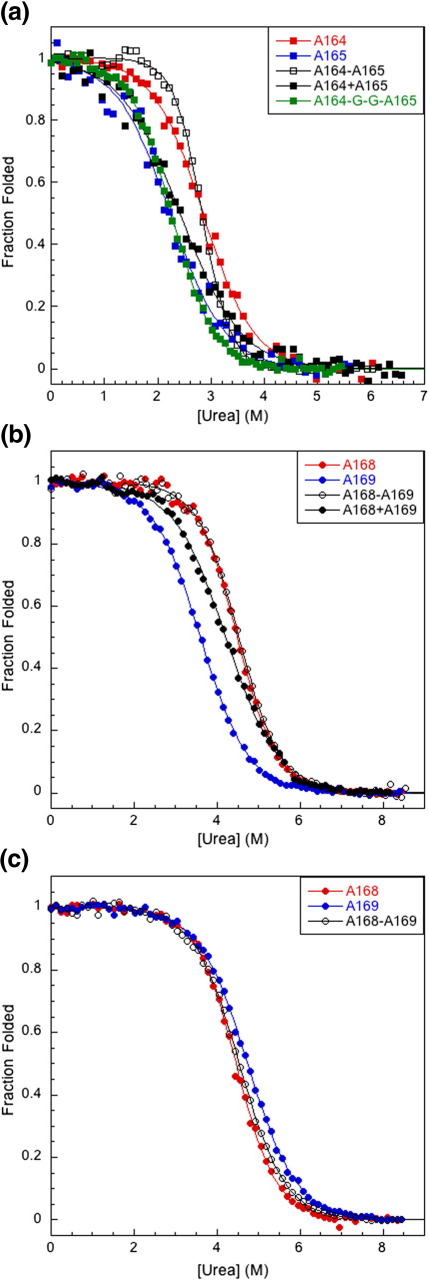
Equilibrium data. (a) A164 (red filled squares), A165 (blue filled squares), A164–A165 (black open squares), and A164–Gly–Gly–A165 (green filled squares) in low-salt buffer (10 mM phosphate, pH 7.0, and 150 mM NaCl). The apparent *m* value of A164–A165 is approximately twice that of the isolated domains. Compare with the equilibrium curve for an equimolar mixture of A164 and A165 (black filled squares); note that these equilibrium data are shifted towards A165 due to the fluorescence signal of A165 being approximately three times that of A164. Also note that the apparent *m* value of the A164–Gly–Gly–A165 mutant is no longer twice that of the isolated domains, indicating a loss of cooperativity at equilibrium. (b) A168 (red filled circles), A169 (blue filled circles), and A168–A169 (black open circles) in low-salt buffer (10 mM phosphate, pH 7.0, and 0 M NaCl). The [urea]_50%_ of A169 increases to that of A168 in the tandem, suggesting that the domains are interacting in A168–A169. Compare with the equilibrium curve for an equimolar mixture of A168 and A169 (black filled circles). (c) A168, A169, and A168–A169 in high-salt buffer (10 mM phosphate, pH 7.0, and 500 mM NaCl). The [urea]_50%_ of A169 is slightly higher than that of A168. The [urea]_50%_ value of the tandem lies approximately midway between that of A168 and A169, as would be expected if the domains were not interacting.

A164 is somewhat more stable than A165 (midpoints of denaturation, [urea]_50%_, of 2.8 M and 2.2 M, in 150 mM NaCl, respectively) (Fig. 2a; Table 1). As is expected for two similar structures, the *m* values for the two domains are similar (1.3 and 1.2 kcal mol^− 1^ M^− 1^ in 150 mM NaCl, respectively). The unfolding of the tandem protein A164–A165 reveals a single transition, with an apparent [urea]_50%_ the same as that of A164 (2.8 M) but very importantly with an apparent *m* value of almost twice that of the single domains (2.2 kcal mol^− 1^ M^− 1^). This is indicative of a system where the two domains are unfolding as a single cooperative unit with no significant accumulation of intermediates during equilibrium unfolding.^1^ Note that the result is very different from that obtained when the two domains are mixed in equimolar amounts (Fig. 2a). Addition of 500 mM NaCl has a destabilizing effect on the isolated domains and on the tandem but does not affect cooperativity—the tandem still unfolds in an all-or-none manner, with an increased apparent *m* value (Table 1). A variant of A164–A165 was generated, by insertion of two Gly residues within the β-strand connecting the two domains, between Gln31550 and Ala31551. This variant has a reduced apparent [urea]_50%_ value, but more importantly, the apparent *m* value is significantly lower (1.5 kcal mol^− 1^ M^− 1^) and is no longer twice that of the isolated domains (Fig. 2a; Table 1).

### Domains in A168–A169 do not behave as a cooperative unit at equilibrium

In 10 mM phosphate, pH 7.0, A168 is more stable than A169 ([urea]_50%_ of 4.5 and 3.6 M, respectively), but again the *m* values of the individual domains are similar (1.2 and 1.0 kcal mol^− 1^ M^− 1^, respectively; Fig. 2b; Table 1). In this low-salt buffer, there is a single equilibrium transition for A168–A169 that resembles almost exactly that of A168, and very importantly, the apparent *m* value is the same as that for a single domain (1.2 kcal mol^− 1^ M^− 1^). [Note that the CD/fluorescence data (not shown) clearly demonstrate that both domains are initially fully folded in the tandem protein.] This is significantly lower than the apparent *m* value expected if the protein behaved as a single cooperative folding unit. The apparent [urea]_50%_ for A168–A169 is the same as that for the more stable domain, A168, and higher than that obtained when the same experiment was performed on an equimolar mixture of the two isolated domains (Fig. 2b). A169 is apparently stabilized by A168. Importantly, however, in the presence of 500 mM NaCl, there is no longer stabilization of A169 in the tandem protein. A169 is stabilized in high salt and the apparent [urea]_50%_ value of the tandem lies approximately midway between that of each single domain, as expected for a non-covalent mixture of domains (Fig. 2c; Table 1).

### Kinetics of A164, A165, and A164–A165

**Fig. 3:**
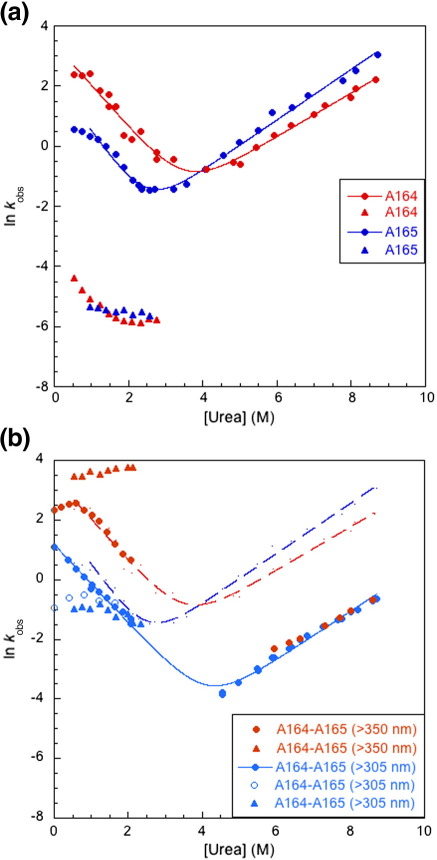
Kinetics of A164, A165, and A164–A165 (in 10 mM phosphate, pH 7.0, and 150 mM NaCl). (a) Observed rate constants for A164 (red) and A165 (blue) alone. Both domains have two folding phases, one fast (filled circles) and one slow (filled triangles) phase, attributed to proline isomerization. A164 has a single fast unfolding phase. A second, slow unfolding phase (amplitude approximately 10%) was observed for A165 (data not shown); we attribute this to the presence of a misfolded (possibly strand-swapped)^25^ species, although none was detected by analytical size-exclusion chromatography (data not shown). (b) Observed rate constants for A164–A165 compared with the single domains. The fits of the chevron plots for the isolated domains from (a) are shown as a red dashed line for A164 and as a blue dashed line for A165. All data shown in orange represent the rate constants associated with A164 (monitored at wavelengths > 350 nm); all data shown in cyan represent the rate constants for A165 (monitored at wavelengths > 305 nm). All data below 3 M urea represented by circles were collected by double-jump (interrupted unfolding) experiments: orange filled circles and cyan open circles were collected using an unfolding delay time of 60 s; cyan filled circles were collected using an unfolding delay time of 5 s. All data below 3 M urea represented by triangles and above 4 M urea represented by circles were collected by single-jump experiments. The data representing slow phases (attributed to proline isomerization) are not shown. The presence of rollover < 1.5 M urea in A164–A165 suggests that when both domains are unfolded, an intermediate slows the folding of both domains; this may be a misfolded intermediate, reflected in the increase in folding rate constant *versus* urea, observed for both domains at very low denaturant concentrations.

All these Ig domains contain a native *cis*-proline. Both A164 and A165 have a fast and a slow refolding phase (Fig. 3a); the slow refolding phases are attributed to proline isomerization and account for > 80% of the overall fluorescence amplitude (Supplementary Fig. S1a). Refolding of A164–A165 was studied using a combination of single-jump and double-jump experiments (Fig. 3b). In single jump, three refolding phases were observed, the slowest attributed to proline isomerization (data not shown). The middle phase reports on the refolding of A165, and there is significant rollover below 1 M urea. The fastest refolding phase cannot be attributed to the folding of either domain and is denaturant independent. In interrupted unfolding experiments, however, the results are somewhat clearer: with a  5 s delay a single refolding phase corresponding to A165 can be clearly detected, without the rollover seen in the single-jump experiments. With a longer delay (60 s) two refolding phases are seen, each corresponding to one of the single domains (Fig. 3b). However, with the longer delay, we again see rollover below 1 M urea.

A165 unfolds somewhat faster than A164, but for A164–A165, only a single unfolding phase is observed, with rate constants significantly lower than those of the individual domains (Fig. 3b). A164 and A165 have different fluorescence properties, and so unfolding was monitored using both a  305-nm cutoff filter (where the major signal change comes from A165) and a  350-nm cutoff filter (where the major signal change comes from A164). Interestingly, both gave a single unfolding phase with the same rate constants, but an amplitude was only detectable at higher urea concentrations (> 6 M) for the  350-nm cutoff experiments.

### Kinetics of A168, A169, and A168–A169

**Fig. 4:**
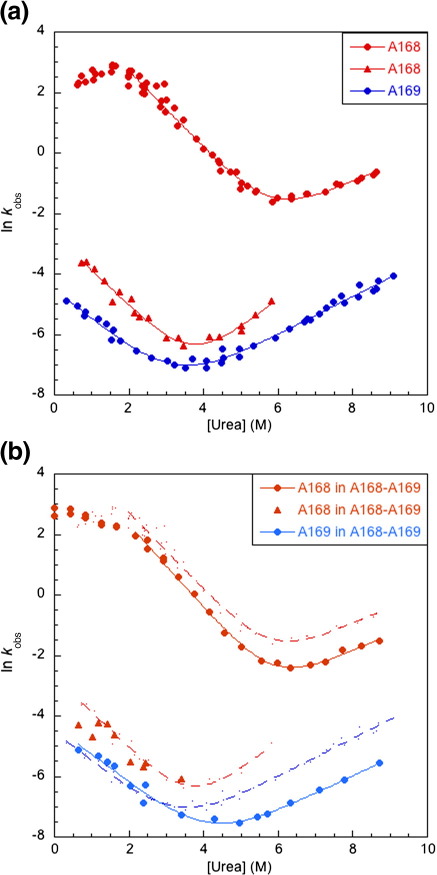
Kinetics of A168, A169, and A168–A169 (in 10 mM phosphate, pH 7.0). (a) Observed rate constants for A168 and A169 alone. A168 has two folding phases, one fast phase (red filled circles) and one slow, proline-isomerization-limited phase (red filled triangles). A169 has a single slow phase (blue filled circles). (b) Observed rate constants for A168–A169 compared with the single domains. Orange filled circles represent the fast phase (due to the folding of the A168 domain); orange filled triangles represent a slow phase associated with the A168 proline-isomerization-limited phase; cyan filled circles represent the slow phases associated with folding and unfolding of A169. The fits of the chevron plots for the isolated domains from (a) are shown as red broken lines for A168 and a blue broken line for A169.

The kinetics of folding and unfolding of A168–A169 and its separate constituent domains were investigated in low-salt buffer. A168 has a fast and a slow refolding phase and a single fast unfolding phase (Fig. 4a); we estimate that the fast phase accounts for ∼ 20% of the amplitude (Supplementary Fig. S1b). Interrupted unfolding experiments confirm that the slow refolding phase is due to proline isomerization (data not shown). Rollover observed below 2 M urea, in the fast refolding phase of A168, was independent of protein concentration, which is indicative of the presence of an intermediate. For A169, there is a single slow refolding and unfolding phase; no faster phase can be detected. We infer that the refolding rate constant of A169 is the same as, or slower than, the rate constant of proline isomerization (Fig. 4a).

A168–A169 has both fast and slow refolding phases (Fig. 4b). The fast folding phase is low in amplitude; hence, the data were collected using interrupted unfolding experiments. This fast phase, which shows protein-concentration-independent rollover below 2 M urea, is apparently due to refolding of A168, being just slightly slower than that for A168 alone. There are also two slow refolding phases that coincide with those of A168 and A169 alone. Two phases are observed for the unfolding of A168–A169; both of these are significantly lower than the unfolding phases of the single domains.

## Discussion

### The equilibrium behavior of the two tandem proteins is very different

We have previously demonstrated that equilibrium *m* values are absolutely key in deciding whether the domains in a multidomain protein are folding independently of each other or behaving as a cooperative, all-or-none unit.^1,16,22^ A164–A165 acts as a cooperative folding unit at equilibrium; we see a single transition, but most importantly, the apparent *m* value for this transition is nearly double that of the isolated domains (an *m* value reflects the change in solvent-accessible surface area upon unfolding and so is related to the size of the protein).^26^ In tandem repeats of identical domains that unfold independently, the apparent *m* value is the same as that of the individual domains.^1^ We infer that there are stabilizing interactions between A164 and A165 in the tandem protein; thus, when one domain unfolds the second domain becomes less stable and also unfolds. Thus, no significant population of an intermediate species, with one domain folded and the other unfolded, will accumulate. Our results suggest that the mutually stabilizing interactions are present at the interface in A164–A165, as disruption of the linking β-strand, by insertion of two Gly residues, results in a loss of cooperativity.

A168–A169 is a more complicated system. Our equilibrium experiments at high salt show that A168–A169 behaves exactly as has been predicted for a noninteracting system where the two domains are independent of each other. Where the denaturation midpoints are close, one expects to see a denaturation midpoint for the tandem between the two individual domains and an apparent *m* value that is close to or slightly lower than that of the individual domains. However, at low salt, we see very different behavior from that seen for a mixture of the two domains. The midpoint for the tandem is the same as that for the most stable domain (A168) and not midway between the two domains, as is seen for the mixture. Again, the apparent *m* value is the same as that for a single domain; hence, we definitively say that the two domains are not unfolding in an all-or-none fashion. We have the conundrum that, in the tandem protein, A169 is apparently stabilized by A168 at low salt but not at high salt—a possible explanation for this is explored later.

### The folding pathway of A164–A165

The analysis of the folding kinetics of A164–A165 is facilitated by the different fluorescence behavior‡. If we monitor wavelengths > 305 nm (using a cutoff filter in the stopped-flow spectrophotometer), we observe a gain in fluorescence upon folding that is attributable to A165. With a cutoff filter of 350 nm, we observe a loss of fluorescence on refolding, largely attributable to A164. In the isolated domains, A164 folds faster than A165. In interrupted unfolding experiments on tandem A164–A165, we see two refolding phases. The faster phase (only detected using a  350-nm cutoff filter) has the same rate constants as A164. The slower phase (only detected using the 305-nm filter) is associated with rate constants very slightly slower than A165 alone. Thus, in the tandem, we see A164 folding first, in the presence of unfolded A165, with the same rate constant as A164 alone. A165 then folds with folding slowed very slightly by the presence of folded A164.

We see only a single unfolding phase for A164–A165 irrespective of the observation wavelength. In kinetics, if there are two interdependent phases, and the slow phase precedes the fast phase, then only the slow phase is observed. There are two reasons for supposing that the rate-limiting step in the unfolding of A164–A165 is the unfolding of the A165 domain, in the presence of still folded A164. First, in the isolated domains, A165 unfolds more rapidly than A164. If there are stabilizing interactions between the two domains at the interface in the native state of the tandem, then the two domains will be stabilized to the same extent and the relative rates of unfolding should remain the same—that is, A165 should unfold before A164 in the tandem. Second, when using the  350-nm cutoff filter, no unfolding is observed below ∼ 6 M urea—that is, rate constants below this value must reflect the unfolding of A165. Thus, the data suggest that in unfolding, A165 unfolds first and then the A164 domain, which is no longer stabilized by folded A165, unfolds much faster, probably with the same rate constant as A164 alone.

**Fig. 5:**
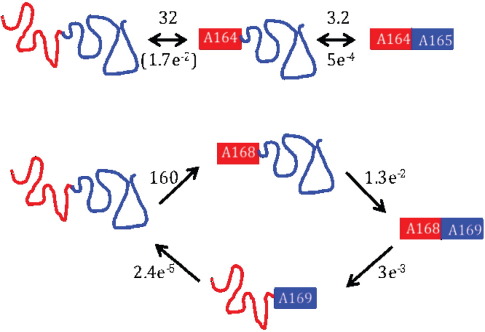
Folding and unfolding pathways of the tandem proteins. All rate constants for the transitions are given in s^− 1^. Top: A164–A165; the folding and unfolding pathways are the reverse of each other: the folding intermediate comprises folded A164 and unfolded A165. In parentheses: this rate constant could not be measured directly; thus, we are assuming that unfolded A165 does not affect the stability of A164, and the unfolding rate constant is the same as that for A164 alone. Bottom: Since the rate constants for folding of A168 and A169 are very different, A168 both folds and unfolds faster than A169 in the tandem protein. Thus, the folding and unfolding intermediates are different species.

Thus, the kinetic pathway for A164–A165 folding proceeds via an intermediate with A164 folded and A165 unfolded (Fig. 5). We can compare the chevron plot for the A165 domain alone with A165 in the presence of folded A164 (Fig. 3b): first, note that the midpoint of A165 in the tandem is similar to that of A164 alone; this supports our proposed mechanism for the cooperative behavior we observe at equilibrium. Secondly, we can estimate that A165 is stabilized by approximately 2 kcal mol^− 1^ by the folded A164 domain. Since we cannot observe the A164 domain folding in the presence of folded A165, we cannot quantify the effect of A165 on A164. A164 and A165 are marginally stable domains (Δ*G* < 4 kcal mol^− 1^ and < 3 kcal mol^− 1^, respectively, in 150 mM NaCl); these results show that the interface between these domains contributes significantly to their overall stability.

### The folding pathway of A168–A169

Although the high-salt data suggest clearly that A168 and A169 do not interact in the tandem protein, the low-salt data were less clear. Thus, the kinetics were investigated under low-salt conditions. A168 folds and unfolds significantly faster than A169, both alone and in the tandem protein. Thus, interestingly, the folding and unfolding pathways of this tandem protein are not the reverse of each other and a different intermediate is populated in folding compared with unfolding (Fig. 5). Such behavior, with different folding and unfolding intermediates, has been seen previously.^27^ A168 both folds and unfolds slightly more slowly in the tandem protein. Analysis of the two chevrons representing the fast kinetic phases of A168 alone and in the tandem (Fig. 4b) shows that the midpoint is unaffected; the stability of A168 is the same in the tandem as in the isolated domain. A169 folds at the same rate in the presence of folded A168, but interestingly, it unfolds significantly more slowly when attached to unfolded A168 (Fig. 4b). Comparison of the A169 chevrons suggests that A168 stabilizes A169 by about 1 kcal mol^− 1^—even when A168 is unfolded.

It is well established that if domain boundaries are selected inappropriately and domains are cut too short, then interdomain interactions might be incorrectly attributed.^28^ Inspection of the crystal structure and the high-salt data and comparison with previous studies of Ig-like domains all suggest that we cannot ascribe the apparent stabilization of A169, by unfolded A168, to incorrect boundary selection.^10,23,24^ However, extension of A169 by unfolded A168 will remove the nonnatural, positively charged N-terminus. We note that this is close in space to two basic residues in folded A169: Lys128 in the B–C loop and Arg181 in the E–F turn. It is possible that the interaction between the N-terminus and these positively charged residues is destabilizing A169. This destabilization will be removed on addition of salt, which will shield the charges, or on extension by unfolded A168 (removing the N-terminus). Such a destabilizing effect of an artificially engineered terminal charge has been observed previously.^29^

### Kinetic rate constants explain cooperative behavior at equilibrium

**Fig. 6:**
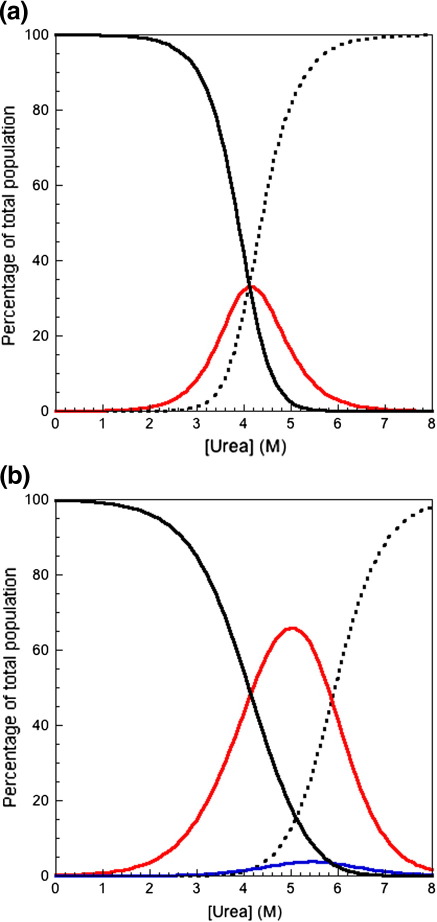
Modeling of the equilibrium populations of the fully folded and fully unfolded tandems and intermediate species determined from the kinetic rate constants and kinetic *m* values. (a) Equilibrium populations of fully folded A164–A165 (black continuous line), fully unfolded A164–A165 (black broken line), and the intermediate A164^F^–A165^U^ (red continuous line). (b) Equilibrium populations of fully folded A168–A169 (black continuous line), fully unfolded A168–A169 (black broken line), the intermediate A168^F^–A169^U^ (red continuous line), and the intermediate A168^U^–A169^F^ (blue continuous line).

The equilibrium populations of the native, intermediate, and denatured species, at different denaturant concentrations, can be modeled from the kinetic rate constants and kinetic *m* values, for both tandem proteins (Fig. 6).^30^ In A168–A169, the intermediate species with A168 folded and A169 unfolded (A168^F^–A169^U^) accumulates to ∼ 70%; there is a much smaller population (∼ 5%) of the intermediate with A168 unfolded and A169 folded (A168^U^–A169^F^) (Fig. 6b). Interestingly, the equilibrium intermediate is the same as the folding (not the unfolding) intermediate and contains folded A168, which is the more stable domain. In A164–A165, the intermediate with A164 folded and A165 unfolded (A164^F^–A165^U^) accumulates to only ∼ 30% (the intermediate with A164 unfolded and A165 folded is not populated at equilibrium) (Fig. 6a). A168^F^–A169^U^ accumulates in the transition region of A168–A169, as the formation of this intermediate from fully unfolded tandem is 4 orders of magnitude faster than its conversion to fully folded tandem. In A168–A169, both rate constants corresponding to each domain are observed in single-jump folding and unfolding experiments. In A164–A165, the formation of A164^F^–A165^U^ from fully unfolded tandem is 1 order of magnitude faster than its conversion to fully folded tandem; however, in unfolding, conversion of A164^F^–A165^U^ to fully unfolded protein is 2 orders of magnitude faster than its formation from folded tandem; thus, a much smaller population of intermediate accumulates in the transition region of A164–A165.

### Explaining the differences between the tandem pairs of domains

Our results on A164–A165 lead us to suppose that the presence of a continuous β-strand between tandem Ig domains imparts cooperative folding, as was reported for the domain/domain-connecting helices in spectrin domain repeats.^31^ Our analysis of A168–A169 demonstrates clearly that this is not the case. As the overall domain/domain arrangement in A164–A165 and A168–A169 is virtually identical, the differences in folding behavior are likely to result from subtle differences within the respective domain/domain interfaces, which we thus investigated in further detail (Fig. 1b and c).

The size of the interface between A164 and A165 is about one-third larger than that between A168–A169 (buried surface areas of 293 Å^2^ and 217 Å^2^, respectively), perhaps explaining, at least in part, why only A164–A165 demonstrates cooperative folding behavior. It is worth noting, however, that neither of these interfaces is particularly large,^13^ which is possibly why the magnitude of the stabilization in A164–A165 is small, ∼ 1–2 kcal mol^− 1^. In both tandems, there is a connecting β-strand with regular β-sheet interactions in both neighboring domains (in cyan in Fig. 1b and c). Only in A164–A165 is there an extensive network of specific, side-chain-mediated, hydrogen-bonding interactions at the domain interface. Most importantly, there is a buried salt bridge between Arg130 and Glu75 at the core of the interface. By contrast, the domain interface of A168–A169 is mainly composed of nonspecific hydrophobic interactions. At the periphery of the interface in A168–A169, there is a surface, solvent-exposed salt bridge between Asp69 and Lys129 (Fig. 1c). We have not characterized the effects of any mutations at the interface of these domains, apart from the Gly insertion mutant (because of the difficulty of producing mutant proteins), but we speculate that the A164–A165 interface is more stabilizing due to the presence of the network of specific side-chain interactions, particularly the buried salt bridge between Glu75 and Arg130. The burial of these domain/domain electrostatic interactions may explain why addition of 500 mM salt does not abrogate the cooperativity in A164–A165.

This comparative analysis has led us to conclude that a domain/domain arrangement, as we have observed for A164–A165 and A168–A169, involving a shared β-strand connecting the adjacent Ig domains and forming a restricted domain/domain interface,^20^ generates an overall domain/domain architecture with a potential for cooperative folding/unfolding. However, the results of our study suggest that the molecular details in terms of presence or absence of specific domain/domain interactions may be crucial in determining to what extent such cooperativity is indeed observed.

Thus, predicting the architecture of titin and of similar tandem repeat proteins from sequence comparisons is not simple. In particular, where interfaces between domains are small, the effects may be subtle. Careful structural analysis, combined with biophysical studies, is needed to elucidate the structure and properties of multidomain proteins.

## Materials and Methods

### Protein expression and purification

The genes encoding A168, A169, and A168–A169 were inserted into a modified version of the vector pRSETA (Invitrogen), which encodes an N-terminal histidine tag. Protein expression was carried out in *Escherichia coli* C41 cells,^32^ and the protein was purified from the soluble fraction, after centrifugation, by affinity chromatography on Ni^2+^-agarose resin (Qiagen). The bound protein was cleaved from the resin with thrombin and further purified by gel filtration using a G75 Superdex 26/60 column (GE Healthcare) in 10 mM phosphate buffer, pH 7.0, and 150 mM NaCl. The purified protein was dialyzed into 10 mM phosphate, pH 7.0, and stored at 4 °C in this buffer supplemented with 0.2 mM TCEP. The genes encoding A164, A165, and A164–A165 were cloned into a pETZ2-1a vector containing an N-terminal histidine tag, a z2 carrier, and a tobacco etch virus protease cleavage site; the mutant A164–G–G–A165 was generated using the Phusion Site-Directed Mutagenesis Kit (Stratagene). Protein expression was carried out in *E. coli* BL21(DE3) cells, and the protein was purified from the soluble fraction, after centrifugation, by affinity chromatography on Ni^2+^-agarose resin (Qiagen). Bound protein was eluted in 25 mM Tris, pH 7.5, supplemented with 300 mM NaCl and 400 mM imidazole. The eluted protein was digested with tobacco etch virus protease and dialyzed into 25 mM Tris, pH 7.5, and 300 mM NaCl. A second Ni^2+^-agarose step removed the cleaved solubility tag, and a final purification step was performed using a Hi-Load 16/60 Superdex 75 column (GE Healthcare).

### Experimental buffers

To try to establish whether any effects could be directly attributed to surface electrostatic interactions, we investigated the equilibrium behavior of the proteins under both low-salt and high-salt conditions. Note, however, that A165 was particularly aggregation prone and so data (equilibrium and kinetics) for A164, A165, and tandem A164–A165 were collected in buffer containing 150 mM NaCl (rather than 0 M NaCl, as for A168, A169 and tandem A168-A169) as low-salt buffer. For all proteins, the high-salt buffer contained 500 mM NaCl. All kinetics were investigated in low-salt buffer. There are Cys residues in domain A164 (2), A168 (1), and A169 (1); hence, all buffers also contained 5 mM DTT.

### Equilibrium studies

Equilibrium studies of all the proteins were performed in urea, using between 0.5 and 2 μM protein in phosphate buffer, pH 7.0, at 25 °C with the samples being allowed to equilibrate for at least 4 h. The equilibrium behavior of all the proteins was investigated with unfolding monitored by the change in intrinsic fluorescence, on a Varian Cary Eclipse fluorimeter; the excitation wavelength was 280 nm in all cases. Emission was followed at 320 nm for all proteins except A164, which was followed at 360 nm. The data for the isolated domains and for the tandem proteins were fitted to an equation that describes a two-state transition.^33^

### Refolding and unfolding kinetics

Rate constants > 0.005 s^− 1^ were measured using an Applied Photophysics stopped-flow fluorimeter. Experiments were performed with excitation at 280 nm and emission monitored at wavelengths > 320 nm for A168, A169, and A168–A169; > 350 nm for A164 and A164–A165; and > 305 nm for A165 and A164–A165. The experiments on A164, A165, and A164–A165 were carried out in 10 mM phosphate, pH 7.0, and 150 mM NaCl with 5 mM DTT. The experiments on A168, A169, and A168–A169 were carried out in 10 mM phosphate, pH 7.0, and 5 mM DTT. For A168, A168–A169, and A164–A165, double-jump (interrupted unfolding) experiments were performed: 6 μM protein was unfolded in 25 mM NaOH in the appropriate buffer; after an unfolding delay time ranging from 1 to 500 s, the unfolded protein was refolded in 5 mM HCl in the appropriate buffer (with urea), using a 5:1 ratio of acid:alkali. A final concentration of 0.5 to 2 μM protein was used in all stopped-flow experiments, except for refolding experiments investigating the dependence of the refolding rate constant upon protein concentration below 2 M urea in A168 and A168–A169, where a final concentration of 0.2, 1, and 5 μM protein was used. The stopped-flow apparatus was maintained at 25(± 0.5) °C. Rate constants below 0.005 s^− 1^ were determined by monitoring the change in fluorescence, after manual mixing of protein and denaturant, in a Varian Cary Eclipse fluorimeter. Excitation was at 280 nm and emission was monitored in a  1 cm path-length cuvette thermostatted at 25 °C. For both methods, the data from between 3 and 10 experiments were averaged for each denaturant concentration. Data were fitted to equations describing a single exponential, a single exponential with a baseline drift term, or a double exponential, using KaleidaGraph (Synergy Software).

## Figures and Tables

**Table 1:** Equilibrium data

Protein	Δ*G*_D–N_ (kcal mol^− 1^)	[urea]_50%_ (M)	*m*_D–N_ (kcal mol^− 1^ M^− 1^)	[NaCl] (mM)
A164	3.71	2.85	1.30	150
A165	2.68	2.23	1.20	150
A164–A165^a^	6.28^a^	2.83^a^	2.22^a^	150
A164–G–G–A165^a^	3.34^a^	2.26^a^	1.48^a^	150
A164	3.46	2.60	1.33	500
A165	1.51	1.31	1.15	500
A164–A165^a^	5.29^a^	2.63^a^	2.01^a^	500
A168	5.51	4.48	1.23	0
A169	3.70	3.60	1.03	0
A168–A169^a^	5.39^a^	4.53^a^	1.19^a^	0
A168	5.34	4.45	1.20	500
A169	4.72	4.72	1.00	500
A168–A169^a^	4.90^a^	4.54^a^	1.08^a^	500

Tandem A164–A165 displays cooperative behavior at equilibrium (has a high apparent equilibrium *m* value) but A168–A169 does not. The data show that cooperativity in the tandem proteins (as manifested by apparent equilibrium *m* value) is unaffected by salt concentration. For clarity, errors are not shown in the table. The error in free-energy measurements is in the range  ±  0.1–0.2 kcal mol^− 1^.

a The equilibrium data for the tandem proteins were fitted to a two-state equation. However, the tandem proteins are not true two-state systems at equilibrium; thus, Δ*G*_D–N_, [urea]_50%_, and *m* values of A164–A165, A164–G–G–A165, and A168–A169 are all only “apparent” values.

## Footnotes

‡ In this analysis of the refolding of A164–A165, we ignore the slow proline isomerization phase, which is only observed in the single-jump kinetics starting from fully unfolded protein.

## References

[1] Batey S., Nickson A.A., Clarke J. (2008). Studying the folding of multidomain proteins. HFSP J..

[2] Levitt M. (2009). Nature of the protein universe. Proc. Natl Acad. Sci. USA.

[3] Bjorklund A.K., Light S., Sagit R., Elofsson A. (2010). Nebulin: a study of protein repeat evolution. J. Mol. Biol..

[4] Bjorklund A.K., Ekman D., Elofsson A. (2006). Expansion of protein domain repeats. PLoS Comput. Biol..

[5] Tskhovrebova L., Trinick J. (2010). Roles of titin in the structure and elasticity of the sarcomere. J. Biomed. Biotechnol..

[6] Garcia T.I., Oberhauser A.F., Braun W. (2009). Mechanical stability and differentially conserved physical–chemical properties of titin Ig-domains. Proteins.

[7] Hsin J., Strumpfer J., Lee E.H., Schulten K. (2011). Molecular origin of the hierarchical elasticity of titin: simulation, experiment, and theory. Annu. Rev. Biophys..

[8] Improta S., Krueger J.K., Gautel M., Atkinson R.A., Lefevre J.F., Moulton S. (1998). The assembly of immunoglobulin-like modules in titin: implications for muscle elasticity. J. Mol. Biol..

[9] Lee E.H., Hsin J., von Castelmur E., Mayans O., Schulten K. (2010). Tertiary and secondary structure elasticity of a six-Ig titin chain. Biophys. J..

[10] Scott K.A., Steward A., Fowler S.B., Clarke J. (2002). Titin; a multidomain protein that behaves as the sum of its parts. J. Mol. Biol..

[11] Head J.G., Houmeida A., Knight P.J., Clarke A.R., Trinick J., Brady R.L. (2001). Stability and folding rates of domains spanning the large A-band super-repeat of titin. Biophys. J..

[12] Steward A., Adhya S., Clarke J. (2002). Sequence conservation in Ig-like domains: the role of highly conserved proline residues in the fibronectin type III superfamily. J. Mol. Biol..

[13] Han J.H., Batey S., Nickson A.A., Teichmann S.A., Clarke J. (2007). The folding and evolution of multidomain proteins. Nat. Rev., Mol. Cell Biol..

[14] Feige M.J., Hendershot L.M., Buchner J. (2010). How antibodies fold. Trends Biochem. Sci..

[15] Kusunoki H., Minasov G., Macdonald R.I., Mondragon A. (2004). Independent movement, dimerization and stability of tandem repeats of chicken brain alpha-spectrin. J. Mol. Biol..

[16] Batey S., Clarke J. (2006). Apparent cooperativity in the folding of multidomain proteins depends on the relative rates of folding of the constituent domains. Proc. Natl Acad. Sci. USA.

[17] Tskhovrebova L., Trinick J. (2003). Titin: properties and family relationships. Nat. Rev., Mol. Cell Biol..

[18] Tskhovrebova L., Walker M.L., Grossmann J.G., Khan G.N., Baron A., Trinick J. (2010). Shape and flexibility in the titin 11-domain super-repeat. J. Mol. Biol..

[19] Bucher R.M., Svergun D.I., Muhle-Goll C., Mayans O. (2010). The structure of the FnIII Tandem A77–A78 points to a periodically conserved architecture in the myosin-binding region of titin. J. Mol. Biol..

[20] Muller S., Lange S., Gautel M., Wilmanns M. (2007). Rigid conformation of an immunoglobulin domain tandem repeat in the A-band of the elastic muscle protein titin. J. Mol. Biol..

[21] Mrosek M., Labeit D., Witt S., Heerklotz H., von Castelmur E., Labeit S., Mayans O. (2007). Molecular determinants for the recruitment of the ubiquitin-ligase MuRF-1 onto M-line titin. FASEB J..

[22] Batey S., Randles L.G., Steward A., Clarke J. (2005). Cooperative folding in a multi-domain protein. J. Mol. Biol..

[23] Pfuhl M., Improta S., Politou A.S., Pastore A. (1997). When a module is also a domain: the role of the N terminus in the stability and the dynamics of immunoglobulin domains from titin. J. Mol. Biol..

[24] Hamill S.J., Meekhof A.E., Clarke J. (1998). The effect of boundary selection on the stability and folding of the third fibronectin type III domain from human tenascin. Biochemistry.

[25] Hayes M.V., Sessions R.B., Brady R.L., Clarke A.R. (1999). Engineered assembly of intertwined oligomers of an immunoglobulin chain. J. Mol. Biol..

[26] Myers J.K., Pace C.N., Scholtz J.M. (1995). Denaturant *m* values and heat capacity changes: relation to changes in accessible surface areas of protein unfolding. Protein Sci..

[27] Parker M.J., Spencer J., Jackson G.S., Burston S.G., Hosszu L.L., Craven C.J. (1996). Domain behavior during the folding of a thermostable phosphoglycerate kinase. Biochemistry.

[28] Politou A.S., Gautel M., Joseph C., Pastore A. (1994). Immunoglobulin-type domains of titin are stabilized by amino-terminal extension. FEBS Lett..

[29] Meekhof A.E., Hamill S.J., Arcus V.L., Clarke J., Freund S.M. (1998). The dependence of chemical exchange on boundary selection in a fibronectin type III domain from human tenascin. J. Mol. Biol..

[30] Arora P., Hammes G.G., Oas T.G. (2006). Folding mechanism of a multiple independently-folding domain protein: double B domain of protein A. Biochemistry.

[31] Batey S., Scott K.A., Clarke J. (2006). Complex folding kinetics of a multidomain protein. Biophys. J..

[32] Miroux B., Walker J.E. (1996). Over-production of proteins in *Escherichia coli*: mutant hosts that allow synthesis of some membrane proteins and globular proteins at high levels. J. Mol. Biol..

[33] Pace C.N. (1986). Determination and analysis of urea and guanidine hydrochloride denaturation curves. Methods Enzymol..

