# Supplementary material for: Two Immunoglobulin Tandem Proteins with a Linking β-Strand Reveal Unexpected Differences in Cooperativity and Folding Pathways
Source: J Mol Biol. 2012 Feb 10;416(1-5):137–47. doi: 10.1016/j.jmb.2011.12.012 (PMC3277889; doi:10.1016/j.jmb.2011.12.012)
Supplement: Supplementary file 1 — Supplementary materials [file mmc1.pdf]

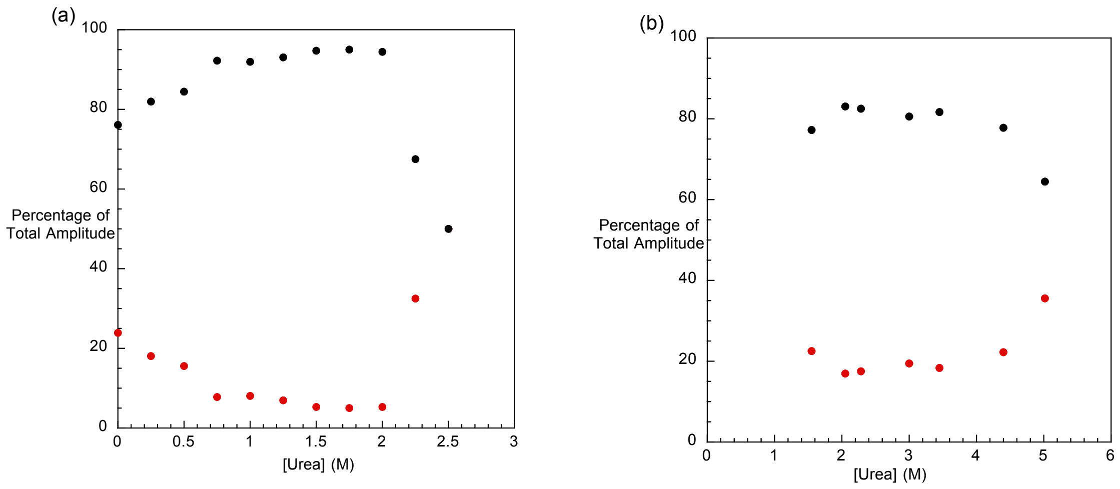

Figure. S1. Relative amplitudes for the fast refolding rate constants (closed red circles) and the slow (*cis* proline limited) refolding rate constants (closed black circles) shown as a percentage of total amplitude, for (a) A164 and (b) A168.

Supplementary Table S1: Position of domain boundaries of the experimental constructs from human cardiac titin (Uniprot accession number, human cardiac titin: Q8WZ42).

Protein	Pdb file	Position
A164	3lcy	31456-31552
A165	3lcy	31549-31649
A164-A165	3lcy	31456-31649
A168	2j8h	31852-31949
A169	2j8h	31946-32049
A168-A169	2j8h	31852-32049
